# Commentary: Impact of integrated care coordination on pediatric asthma hospital presentations

**DOI:** 10.3389/fped.2023.1202602

**Published:** 2023-05-30

**Authors:** Farhad Shaker

**Affiliations:** ^1^ School of Medicine, Tehran University of Medical Sciences, Tehran, Iran; ^2^ School of Public Health, Tehran University of Medical Sciences, Tehran, Iran

**Keywords:** socioeconomic, asthma, integerated care, respiratory disease, pediatric

A Commentary on Impact of integrated care coordination on pediatric asthma hospital presentations By Shaker F. (2023) Front. Pediatr. 11:929819. doi: 10.3389/fped.2022.929819

Dear Editor,

With great interest, i have read the article entitled “Impact of integrated care coordination on pediatric asthma hospital presentations” by Homaira et al. (1), which describes a novel integrative model for managing asthma in pediatrics that has improved clinical outcomes in this population. However, there are some concerns about the reliability and feasibility of this model. Here we have discussed potential obstacles to this model being utilized as a population-based system; moreover, some action to be taken as solutions to the mentioned obstacles have been suggested.

According to the prior investigation (2), performing primary care and developing long-term interactive relationships is more challenging in low SE populations; this accentuates the importance of investigating the effect of integrated care in low SE (socioeconomic) populations, including SEIFA (Socio-Economic Indexes for Areas) decile 1–7, which are in the minority in this study sample. Utilizing the four different indexes of SEIFA, including IRSD (The Index of Relative Socio-economic Disadvantage), IRSAD (The Index of Relative Socio-economic Advantage and Disadvantage), IEO (The Index of Education and Occupation), and IER (The Index of Economic Resources) (3), can provide a better comprehension of the main obstacles that lead to lower efficacy of integrated care in this population.

In addition, an efficient collaboration of different care providers depends on the well-established electronic health record system, which is not available in all countries. As Sadoughi et al. illustrated in their study, some crucial asthma-related data elements are unavailable in Iran's electronic health records, significantly affecting the clinical and management outcomes (4). Concerning this limitation, defining and categorizing an abridged version of essential data that must be available in an information system to perform integrative models can make it more conductible in underdeveloped and developing countries.

Such integrative management should be cost-effective, which is influenced by different factors such as the simplicity of the model, scalability of the target population, and applicability of the model to be implemented as a management program for further indications. Combining currently available integrative models for the different diseases that are similar based on the human resources involved in their management can reduce the overall cost of educating healthcare providers and decrease total infrastructure development. A primary care integrative service for COPD patients has been developed, showing considerable enhancement in the clinical outcome, while no significant increase in health care cost was observed (5). By merging this program with the asthma-related integrative model, a more feasible model can be obtained due to sharing resources for integrative health record system development and defining a more inclusive target population.
